# Supplementary material for: Engineering chirality at wafer scale with ordered carbon nanotube architectures
Source: Nat Commun. 2023 Nov 15;14:7380. doi: 10.1038/s41467-023-43199-x (PMC10651894; doi:10.1038/s41467-023-43199-x)
Supplement: Supplementary file 1 — Supplementary Information [file 41467_2023_43199_MOESM1_ESM.pdf]

Supplementary Information: Engineering Chirality at Wafer Scale with Ordered Carbon Nanotube Architectures

Jacques Doumani[1,2,3], Minhan Lou[3], Oliver Dewey[4,5], Nina Hong[6], Jichao Fan[3], Andrey Baydin[1,7], Keshav Zahn[1], Yohei Yomogida[8], Kazuhiro Yanagi[8], Matteo Pasquali[4,5,7,9,10], Riichiro Saito[8,11,12], Junichiro Kono[1,4,7,10,13] and Weilu Gao[3,4*]

[1]Department of Electrical and Computer Engineering, Rice University, Houston, TX, USA.
[2]Applied Physics Graduate Program, Smalley–Curl Institute, Rice University, Houston, TX, USA.
[3]Department of Electrical and Computer Engineering, The University of Utah, Salt Lake City, UT, USA.
[4]Carbon Hub, Rice University, Houston, TX, USA.
[5]Department of Chemical and Biomolecular Engineering, Rice University, Houston, TX, USA.
[6]J.A. Woollam Co., Inc., Lincoln, NE, USA.
[7]Smalley–Curl Institute, Rice University, Houston, TX, USA.
[8]Department of Physics, Tokyo Metropolitan University, Tokyo, Japan.
[9]Department of Chemistry, Rice University, Houston, TX, USA.
[10]Department of Materials Science and NanoEngineering, Rice University, Houston, TX, USA.
[11]Department of Physics, Tohoku University, Sendai, Japan.
[12]Department of Physics, National Taiwan Normal University, Taipei, Taiwan.
[13]Department of Physics and Astronomy, Rice University, Houston, TX, USA.

*Corresponding author(s). E-mail(s): weilu.gao@utah.edu;

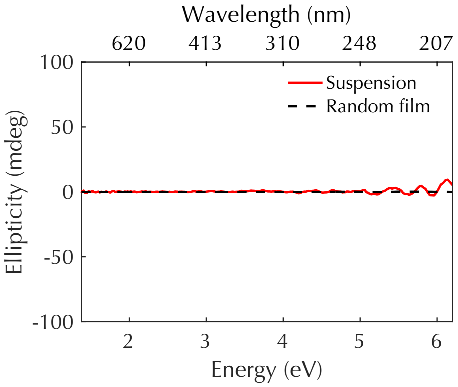

Supplementary Figure 1 CD spectra of the aqueous suspension of CNTs consisting of a racemic mixture (red solid line) and their randomly oriented film prepared via CVF (black dashed line). Source data are provided as a Source Data file.

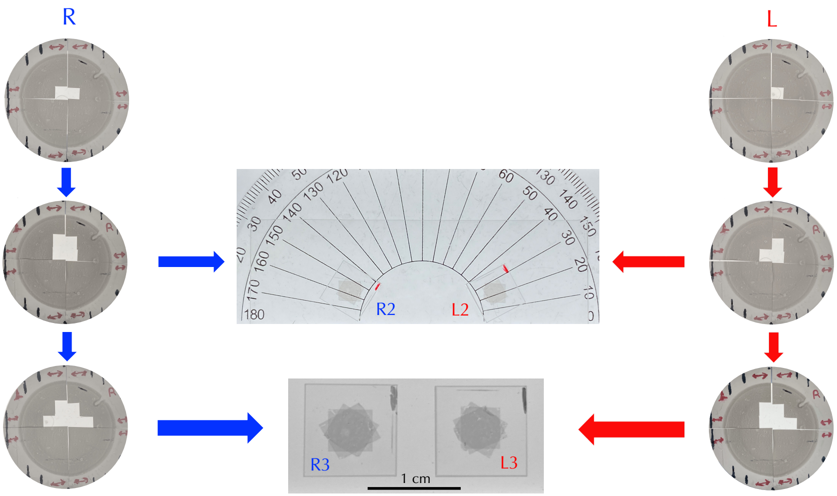

Supplementary Figure 2 Photographs of twist-stacking-produced CNT films in left-handed (red) and right-handed (blue) manners, respectively. The twist angle is 30°.

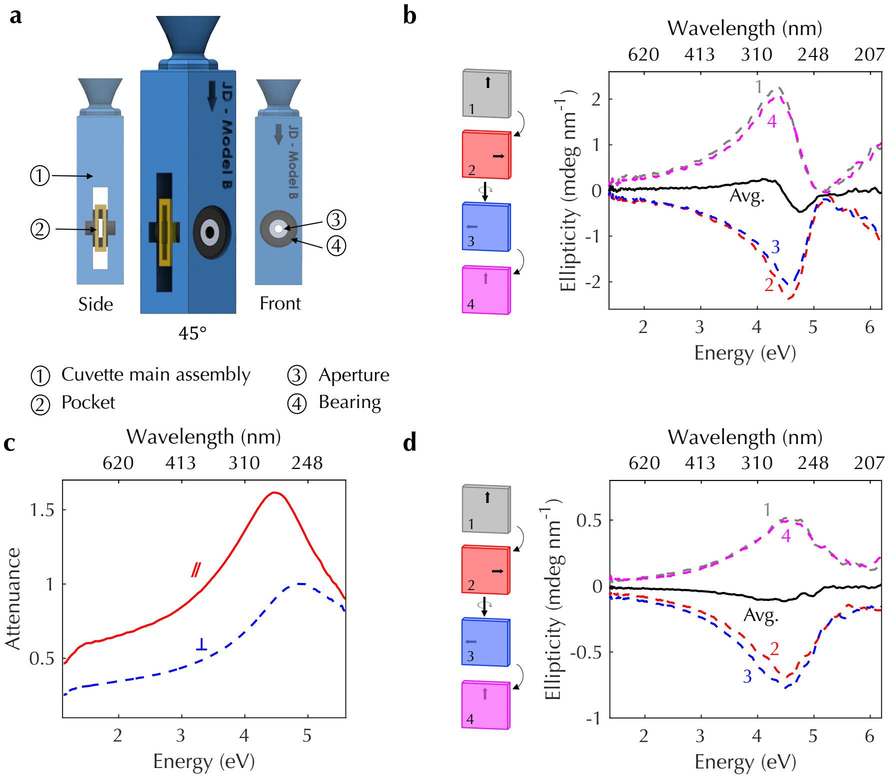

Supplementary Figure 3 Four-configuration CD measurement using a standard CD spectrometer. (a) Schematic of the 3D printing model of the customized sample holder cuvette, which consists of a main assembly, pocket, two apertures, and two bearings for sample rotation. (b) CD spectra for a highly aligned CNT film prepared using CVF under four measurement configurations (grey, red, blue, and pink dashed lines) and their average (black solid line). (c) Linear polarization-dependent attenuation spectra of an aligned CNT film prepared using a shear stress technique. The red solid line is for parallel polarization and blue dashed line is from perpendicular polarization. (d) CD spectra of the aligned CNT film prepared using the shear stress technique under four measurement configurations (grey, red, blue, and pink dashed lines) and their average (black solid line). Source data are provided as a Source Data file.

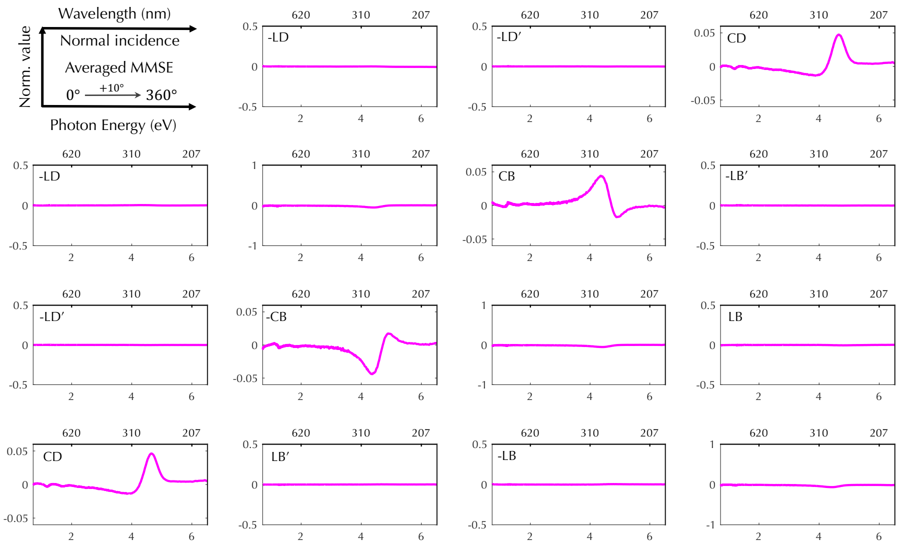

Supplementary Figure 4 Decomposed and averaged Müller matrix spectra for a twisted-stacking-produced twisted 3-layer CNT stack at a twist angle 30°. Source data are provided as a Source Data file.

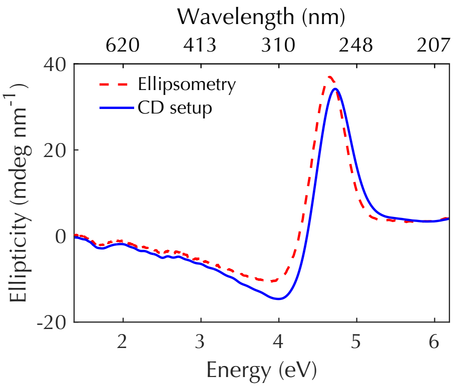

Supplementary Figure 5 CD spectra measured using a CD spectrometer with the four-configuration approach (blue solid line) and spectroscopic ellipsometry (red dashed line) for the sample in Supplementary Fig. 4. Source data are provided as a Source Data file.

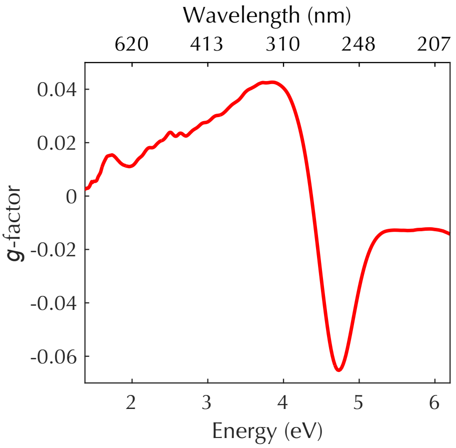

Supplementary Figure 6 The g-factor spectrum for the R3 sample in Fig. 2 of the main text. Source data are provided as a Source Data file.

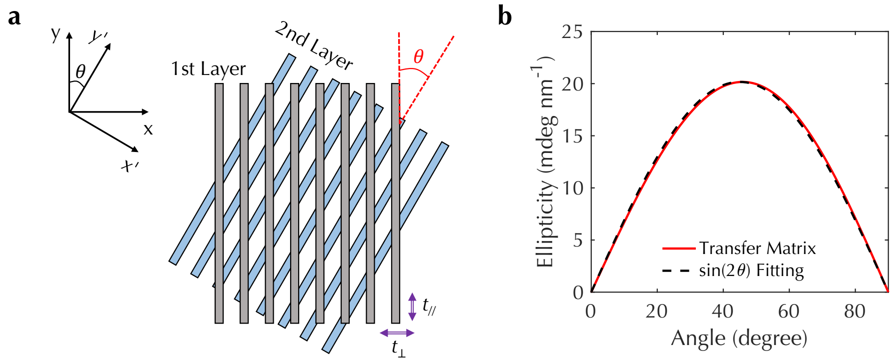

Supplementary Figure 7 Comparison between transfer matrix and Jones calculus methods. (a) Schematic of a twisted two-layer stack of aligned CNTs. (b) The comparison of calculated ellipticity as a function of twist angle using transfer matrix (red solid line) and Jones calculus methods (black dashed line). Source data are provided as a Source Data file.

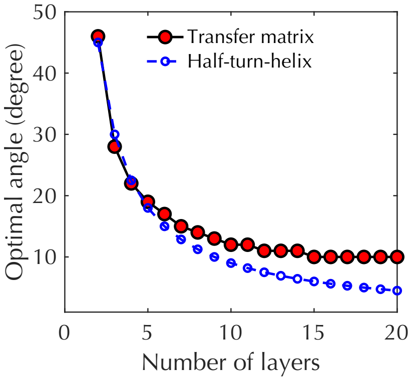

Supplementary Figure 8 Calculated optimal twist angles using transfer matrix method (red markers) and analytical equation (blue markers) as a function of the number of stacked layers for twist-stacking-produced CNT stacks. Source data are provided as a Source Data file.

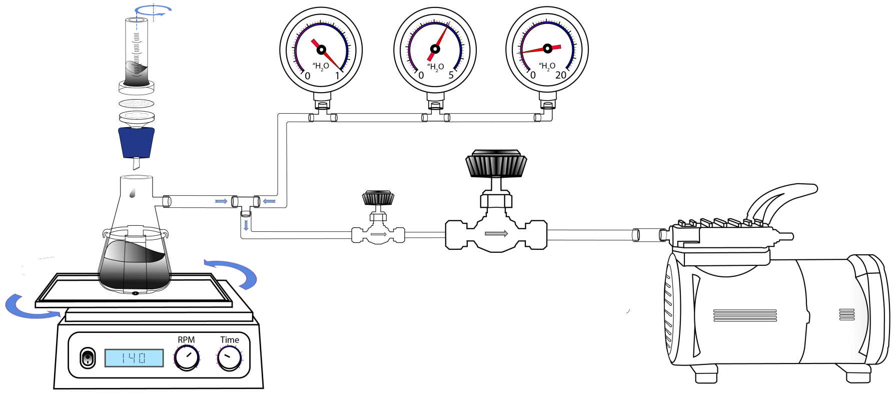

Supplementary Figure 9 Schematic of mechanical-motion-assisted vacuum filtration system that is placed on an orbital shaker. The filtration flow is controlled with three pressure gauges and two valves, which are connected to a vacuum pump.

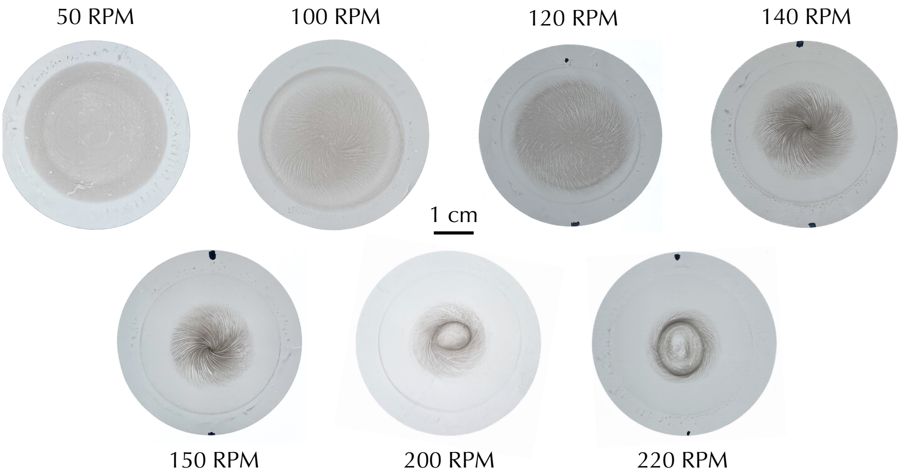

Supplementary Figure 10 Photographs of obtained CNT films through mechanical-rotation-assisted CVF under different rotation speeds.

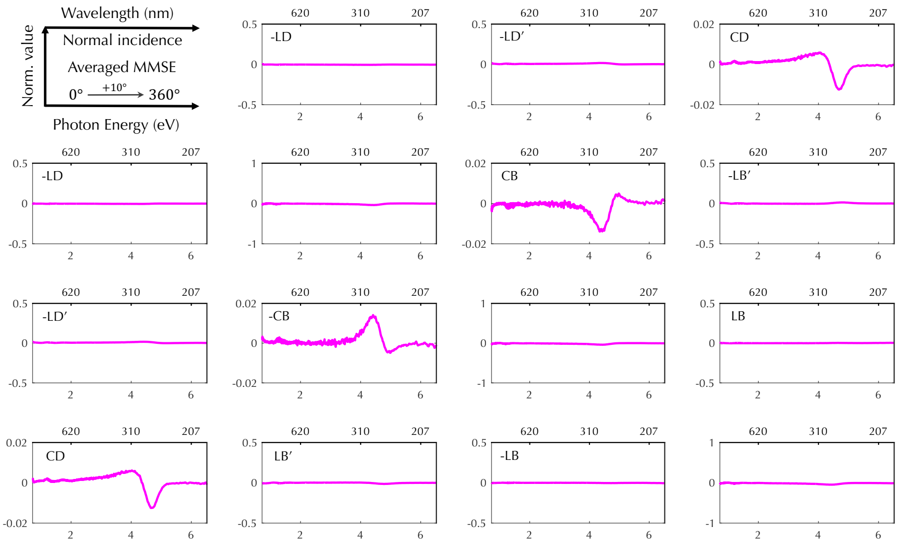

Supplementary Figure 11 Decomposed and averaged Müller matrix spectra for a mechanical-rotation-assisted-CVF-produced CNT film. Source data are provided as a Source Data file.

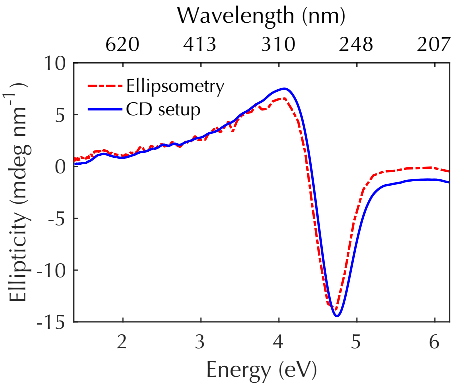

Supplementary Figure 12 CD spectra measured using a CD spectrometer with the four-configuration approach (blue solid line) and spectroscopic ellipsometry (red dashed line) for the sample in Supplementary Fig. 11. Source data are provided as a Source Data file.

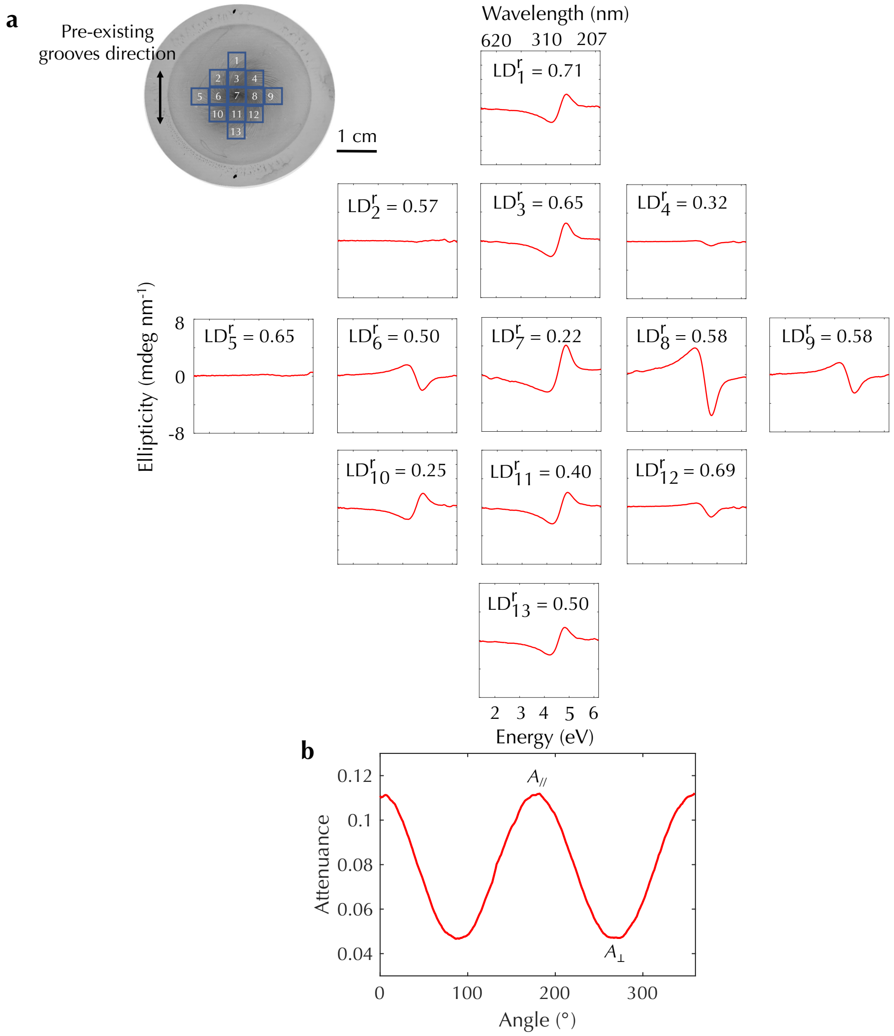

Supplementary Figure 13 Characterization of CNT films prepared using mechanical-rotation-assisted CVF at 140 RPM. (a) CD and reduced linear dichroism (LD^r) mappings. (b) Polarization angle-dependent attenuation for a representative CNT sample, where maximum ($A_{||}$) and minimum attenuation ($A_\perp$) are used to calculate LD^r. Source data are provided as a Source Data file.

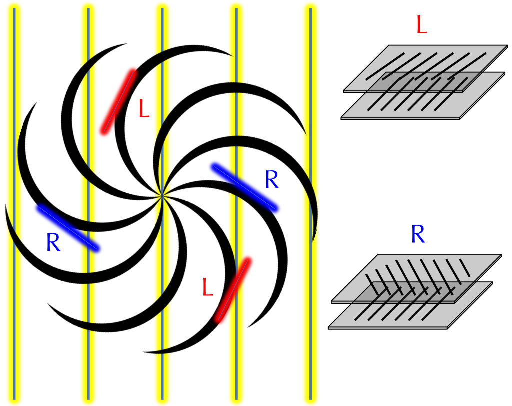

Supplementary Figure 14 Illustration of twist structures in mechanical-rotation-assisted-CVF-produced CNT films with spiral patterns. The yellow lines indicate the direction of bottom aligned CNTs and the black lines indicate a spiral pattern formed on top. There are two different twist configurations depending on the relative rotation of the bottom alignment direction and the local spiral direction.

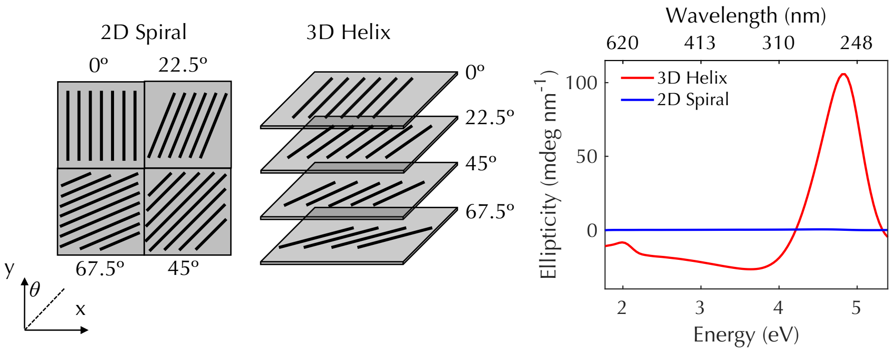

Supplementary Figure 15 Schematic and finite-difference-time-domain simulated CD spectra of CNT films with a 2D spiral pattern (blue line) and a 3D helical structure (red line). Source data are provided as a Source Data file.

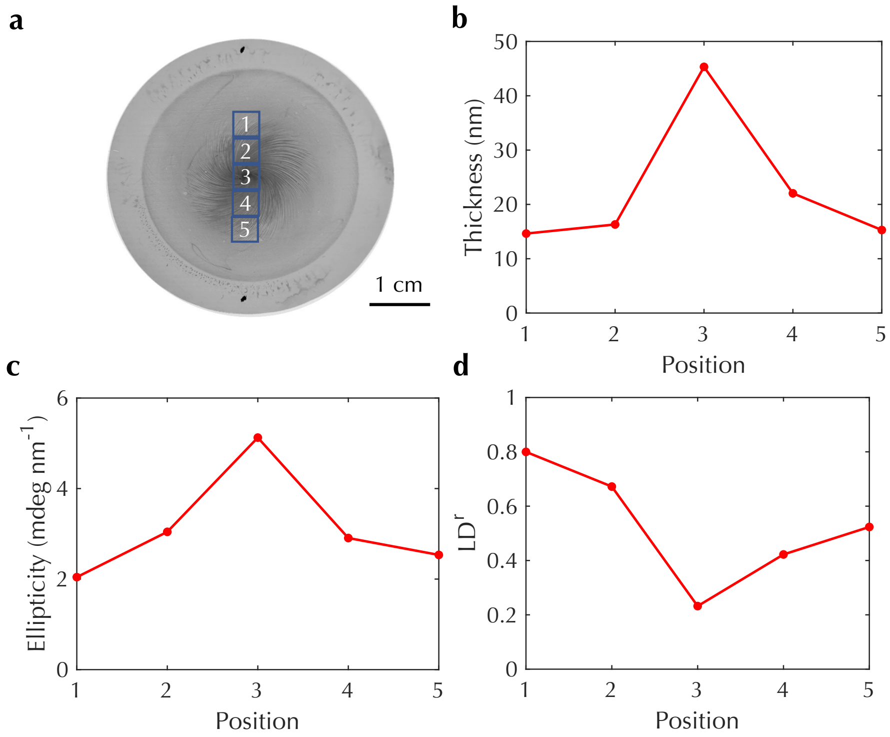

Supplementary Figure 16 Radial mappings of the CNT film in Supplementary Fig. 13. (a) Photograph and radial mappings of (b) thickness, (c) peak CD signal, and (d) LD^r. Source data are provided as a Source Data file.

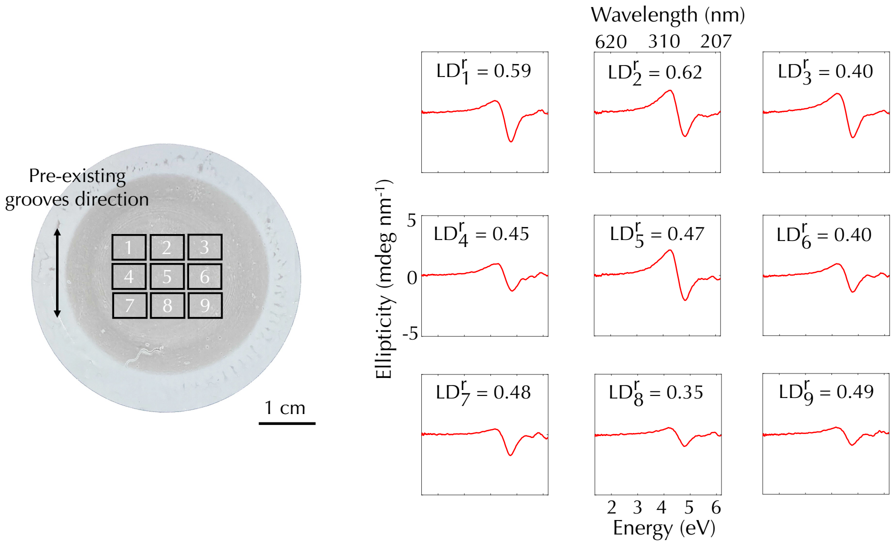

Supplementary Figure 17 CD and reduced linear dichroism (LD^r) mappings of obtained CNT films prepared using mechanical-rotation-assisted CVF at 50 RPM. Source data are provided as a Source Data file.

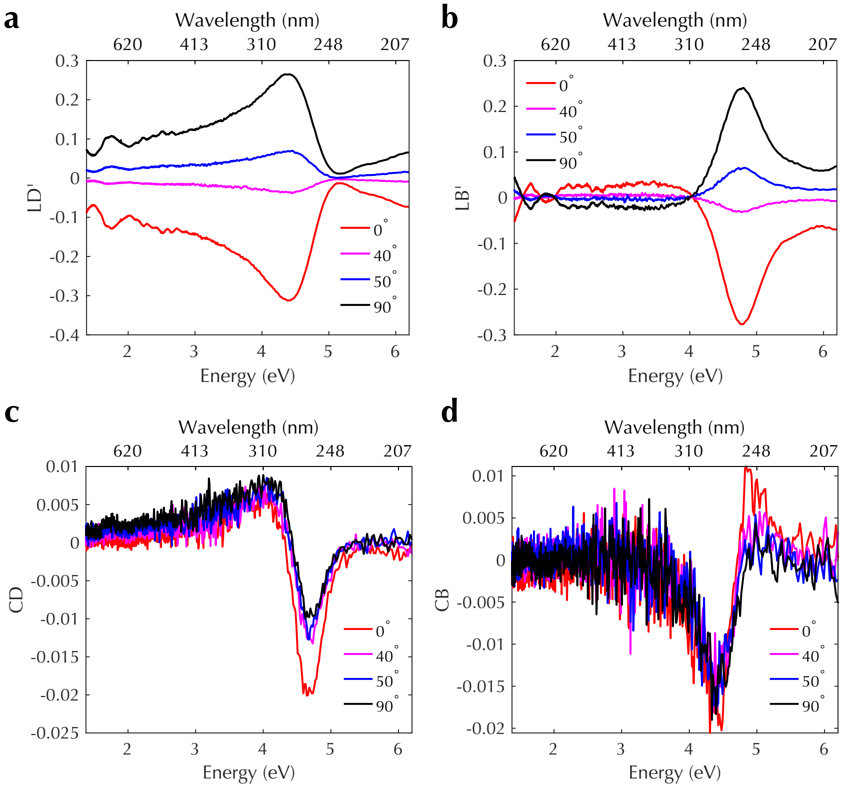

Supplementary Figure 18 In-plane sample rotation angle dependence in spectroscopic ellipsometry. (a) LD' spectra, (b) LB' spectra, (c) CD spectra, and (d) CB spectra under in-plane sample rotation angles of 0° (red lines), 40° (pink lines), 50° (blue lines), and 90° (black lines). Source data are provided as a Source Data file.

Supplementary Table 1 DUV ellipticity for different chiral platforms

Platform	Wavelength (nm)	Ellipticity (mdeg nm^{-1})	g factor	Reference
TiO_2 nanohelix metamaterial	≈270	≈17	≈ 0.05	Ref. [1]
Al collodial chiral plasmonic shell	≈280	≈4	N/A (There is no reported attenuation)	Ref. [2]
Mg nanohelix metamaterial	≈275	≈16	≈ 0.09	Ref. [3]
Al gammadion metamaterial	≈250	≈8.6	N/A (There is no reported attenuation)	Ref. [4]
Twisted bilayer graphene	≈280	≈3	6×10^{-3}	Ref. [5], graphene attenuation from Ref. [6]
$(MBA)_2PbI_4$ perovskites	≈ 220	≈0.2	3×10^{-3}	Ref. [7]
Twist-stacked three-layer CNT films	≈ 260	≈40	≈ 0.07	Our work

Supplementary references

[1] Sarkar, S., Behunin, R. O. & Gibbs, J. G. Shape-dependent, chiro-optical response of uv-active, nanohelix metamaterials. *Nano Lett.* **19** (11), 8089–8096 (2019).

[2] Huang, S. *et al.* Engineering a strong and stable ultraviolet chiroptical effect in a large-area chiral plasmonic shell. *Opt. Express* **30** (17), 31486–31497 (2022).

[3] Jeong, H.-H., Mark, A. G. & Fischer, P. Magnesium plasmonics for uv applications and chiral sensing. *Chem. Comm.* **52** (82), 12179–12182 (2016).

[4] Leite, T. R., Zschiedrich, L., Kizilkaya, O. & McPeak, K. M. Resonant plasmonic–biomolecular chiral interactions in the far-ultraviolet: Enantiomeric discrimination of sub-10 nm amino acid films. *Nano Lett.* **22** (18), 7343–7350 (2022).

[5] Kim, C.-J. *et al.* Chiral atomically thin films. *Nat. Nanotechnol.* **11** (6), 520 (2016).

[6] Chae, D.-H. *et al.* Excitonic fano resonance in free-standing graphene. *Nano Lett.* **11** (3), 1379–1382 (2011).

[7] Lu, H. *et al.* Spin-dependent charge transport through 2d chiral hybrid lead-iodide perovskites. *Sci. Adv.* **5** (12), eaay0571 (2019).
