# Supplementary material for: Engineering chirality at wafer scale with ordered carbon nanotube architectures
Source: Nat Commun. 2023 Nov 15;14:7380. doi: 10.1038/s41467-023-43199-x (PMC10651894; doi:10.1038/s41467-023-43199-x)
Supplement: Supplementary file 3 — Description of Additional Supplementary Files [file 41467_2023_43199_MOESM3_ESM.pdf]

Description of Additional Supplementary Files

File Name: Supplementary Movie 1
Description: Mechanical-rotation-assisted vacuum filtration setup.
